# Widespread Molecular Imprints in the Serum Proteome of COVID-19 Convalescents Uncovering Immune System Sequelae

**DOI:** 10.1016/j.mcpro.2026.101525

**Published:** 2026-02-12

**Authors:** Kun Liu, Zhigang Ren, Bowen Dong, Wenli Liu, Yuyuan Gao, Li Zhang, Jingyi Li, Zhao Sun, Hongyi Li, Qian Zhao, Xinchao Hu, Jinfeng Chen, Yuanyuan Wang, Yang Yang, Lei Zhang, Xinli Xue, Aiguo Xu, Zujiang Yu, Jing-Hua Yang

**Affiliations:** 1 Clinical Systems Biology Laboratories, The First Affiliated Hospital, Academy of Medical Sciences, Zhengzhou University, Zhengzhou, China; 2 Departments of Infectious Diseases, Respiratory and Critical Care Medicine, The First Affiliated Hospital of Zhengzhou University, Zhengzhou, China; 3 Departments of Gastric Intestine and Oncology, The First Affiliated Hospital, Zhengzhou University, Zhengzhou, China

**Keywords:** COVID-19 convalescence, global protein modification, post-translational modification, post-COVID-19 sequelae, proteomics, SARS-cov-2 infection

## Abstract

Post-COVID-19 sequelae have become an emerging global health issue, but the mechanisms for the sustained susceptibility of convalescents to the sequelae remain poorly understood. Here we report the use of a restricted open-search approach to explore the molecular imprints of SARS-CoV-2 infection left on the proteome of 412 COVID-19 patients and convalescences. A total of 827 non-standard amino acid variations, chemically modified residues as well as post-translational modifications, termed non-coded amino acids (ncAAs), are found spreading over 29,814 sites in patient’s serum proteins. Markedly, widespread ncAAs are induced and sustainedly imprinted on the serum proteome predominately perturbing the immunoglobulin-mediated immune response, complement activation and coagulation regulation even 12 months after recovery. Sustained amino acid variations and chemical modifications are found in the complementary‑determining regions (CDRs) of the variable region of immunoglobulin contributing to the interactions between the emerging antibody and antigens; durable chemical amino acid modifications found in the hyper ncAA-modified regions of the constant region of immunoglobulin important for the interaction with the complement and regulatory receptors. In the complement system, inducible ncAAs are memorized in the components essential for the complement activation, amplification cascades and membrane attack processes. Thus, the workflow described in this study can be used to identify the molecular imprints of viral infection at the proteomic scale, particularly the specific antibodies and the immune targets left in COVID-19 patients and convalescents.

## Highlights

• Diverse noncoded amino acids (ncAAs) found in COVID-19 patient serum proteome.
• Persistent amino acid variations in immunoglobulins, complement, and coagulation factors up to 12 months post-COVID-19.
• Serum ncAA imprints reveal immune system sequelae in COVID-19 convalescents.

## Graphical Abstract

**Figure:**
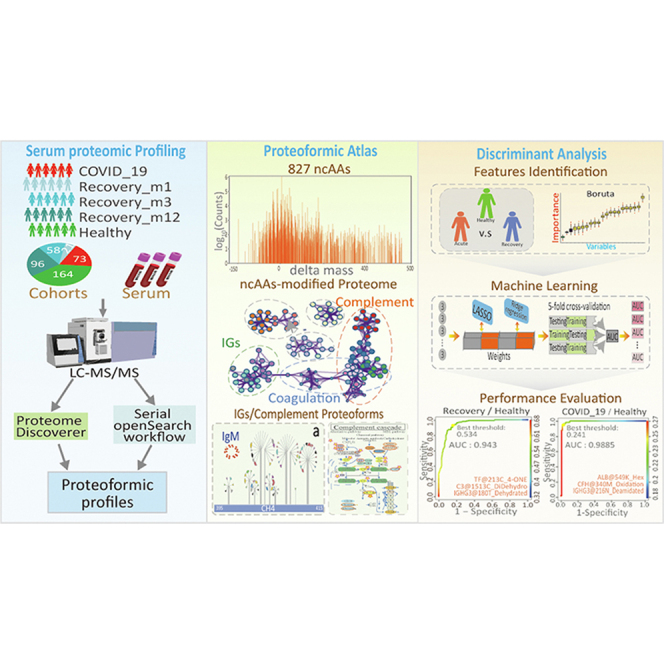

## In Brief

The mechanisms for the sustained susceptibility of COVID-19 convalescents to the sequelae remain poorly understood. Here we report the use of a restricted open-search approach to explore the molecular imprints of SARS-cov-2 infection left on the serum proteome. 827 ncaas are induced and sustainedly imprinted on the serum proteome predominately perturbing the immunoglobulin-mediated immune response, complement activation and coagulation regulation even 12 months after recovery. Workflow in this study identify the molecular imprints of viral infection at the proteomic scale, particularly the specific antibodies and the immune targets left in COVID-19 patients and convalescents.

Although acute symptoms caused by SARS-CoV-2 infection return to baseline health with the SARS-CoV-2 eradication, COVID-19 convalescent individuals may experience severe disorders emerging in neuronal, respiratory, cardiovascular, and immune systems, and irreversible sequelae such as brain atrophy (1, 2, 3, 4, 5, 6, 7, 8), suggesting that SARS-CoV-2 infection may cause persistent or unperceived physiological disorders. However, the mechanisms underlay the susceptibility of convalescent individuals to the sequelae remains unclear (9, 10). There is an urgent need to explore the comprehensive long-term health impact of SARS-CoV-2 infection at the molecular level across COVID-19 course, including recovery.

Human serum proteins alterations have been recognized as indicators of pathophysiological changes in various diseases, including viral infections (11, 12). In acute phase of infection, serological biomarkers including the complement, coagulation, metabolomic molecules have been identified to facilitate the patient’s managements (12, 13, 14, 15). It is reasoned that monitoring virus-left molecular imprints on the proteins at the proteomic scale, as amino acid (AA) derivatives, variations, or modifications in the circulating blood stream (9), provides a viable way to fully understand the entire pathologies of COVID-19, including the onset, progression and recovery stage.

Recently, progresses are made to systematically identify any possible protein modifications and/or variations at the proteomic scale (16, 17, 18). Here, to explore the molecular imprints on proteins that are responsive for the susceptibility of the convalescents to sequelae, we have adapted a sophisticated search workflow and analyzed serum proteins from over 400 COVID-19 patients and convalescents, which uncovers widespread molecular imprints on the serum proteome and a sustained dysregulation of multiple systems including host immune responses, complement, and coagulation.

## Experimental Procedures

### Sample Collection

A total of 412 samples between February, 2019 and March, 2020 were collected in this research. Among them, 73 acute COVID-19 patients, 21 convalescents in 1 month, 58 convalescents in 3 months, and 96 convalescents in 12 months were from Xinyang, Henan; 164 health people were from the First Affiliated Hospital of Zhengzhou University. Both patients currently in the acute phase of COVID-19 and individuals who have recovered from COVID-19 after 1, 3 and 12 months have been diagnosed with severe COVID-19 symptoms by the attending doctors, according to the Chinese Government Diagnosis and Treatment guideline (Edition sixth). These diagnoses are based on fulfilling any of the following three criteria: respiratory distress, respiratory rate of 30 times/min or higher; mean oxygen saturation of 93% or less in a resting state; arterial blood oxygen partial pressure/oxygen concentration of 300 mmHg or less (1 mmHg = 0.133 kPa). Comprehensive demographic and clinical data for both patient cohorts and health controls, assigned unique anonymized identifiers, are provided in Supplemental Table S1, with direct identifiers (*e.g.,* legal names, ID number) removed. All samples included in the study signed informed consent detailing study objectives, benefits, and privacy safeguards. This study was approved by the Ethics Committee of the First Affiliated Hospital of Zhengzhou University (approval number: 2021-Y429-002), adhering to the Declaration of Helsinki and national research ethics guidelines.

The clinical acute phase patients of COVID-19 underwent antiviral therapy with nirmatrelvir/ritonavir, anti-inflammatory treatment with glucocorticoids/tocilizumab, or anticoagulation/antiplatelet therapy. All serum samples were drawn using serum separation tubes (BD 367955), stored at room temperature for approximately 30 min, and then centrifuged at 1500*g* for 10 min. After centrifugation, the serum was collected in new centrifuge tubes and immediately stored at −80 °C.

### Experimental Design and Statistical Rationale

A total of 412 independent serum samples were analyzed to mitigate heterogeneity or instrumental variability. Sample size was determined by power analysis (1−β = 0.8, α = 0.05), ensuring adequate statistical significance false discovery rate (FDR < 0.05) for differential protein quantification. Biological replicates were evaluated using Kruskal-Wallis test for between-group clinical feature differences, followed by permutation-based multiple testing correction to enhance result robustness.

### Sample Preparation for LC-MS/MS

After diluting 10 μl serum with 190 μl 25 mM NH_4_HCO_3_, 1 ml acetone was added to extract the proteins in −40 °C for 1 h. The proteins pellet was acquired by centrifuge at 10,000*g* for 10 min, and subsequently dissolved in 25 mM NH_4_HCO_3_. The protein concentration was measured with a BCA Protein Assay Kit (Beyotime) according the manufacturer’s instructions. Total 3 μg protein was digested with DTT and trypsin (Promega) overnight at 37 °C. Peptides in the supernatant were collected on C18 Zaptip columns, washed with 0.1% formic acid, and eluted with 50% methanol. Desalted peptides were dried in a SpeedVac for LC-MS/MS analysis.

### Mass Spectrometry (MS)

One microgram of trypsinized peptides were dissolved in buffer A (0.1% formic acid) and analyzed using Q Exactive HF-X (Thermo Fisher Scientific) with Data Dependent Acquisition (DDA) mode, scan range within 350 to 2000 m/z in the positive electrospray mode, resolution at 60,000 (@200 m/z) with AGC target of 3e6, and ion injection time (Maximum IT) at 50 ms. Top 20 precursors were selected for the MS/MS experiment. The dynamic exclusion was set to 50 s, and the charge states of peptides were selected as 2 to 7 by using higher energy collisional dissociation. For MS2 spectra, the peptides were analyzed using isolation width of 1.6 m/z in the Orbitrap, normalized higher energy collisional dissociation collision energy (NCE) of 28%, and the AGC target as 5e4 with a resolution at 15,000 and Maximum IT at 45 ms. All the reagents were MS grade and all the analytic time is 120 min.

### Analysis of Delta Masses for Global Protein Modifications

To identify the mass differences between the coding AAs and the actual residues, the acquired MS and MS/MS data were queried against the human UniProt database (Uniprot Proteome ID: UP000005640, Release-2021_04, containing 20,350 unique protein sequences) by an open modification search using the Byonic software (version 1.4; https://www.proteinmetrics.com/products/byonic) (17) spectral alignment algorithm and filtered with Score ≥300, DeltaMods Score ≥10, FDR2D ≤ 0.01 and FDR_uniq.2D ≤ 0.01. Search parameters for Byonic were set including proteolytic enzyme trypsin and allowed for up to two missed cleavage sites. The precursor ion tolerance was set to 10 ppm, the fragment tolerance to 0.6 Da, BlindMode to 1, delta masses from −200 to 500 Da, FDR for peptides to 1%, threshold score to 30, and the probability of the peptides and spectrum match to 0.95. For clustering, delta masses were divided into subgroups with 1 Da intervals bounded by n-0.5 and n + 0.5 Da (n = −200–500). Delta masses in each mass window were analyzed by multivariate clustering using Gaussian mixture components with the following constraints (1): peak half-width >1 ppm (2); peak distance >2 peak widths (3); cluster size >20. Clusters within each window were determined using the Bayesian information criterion, and the outcomes were fitted individually with Gaussian regression to calculate the peak value (expected delta masses), SD, and goodness-of-fit (R^2^). The delta mass clusters reflected the potential protein modifications spanned on the proteome. All MS/MS spectra were scanned by an open-search strategy with pFind3 software (version 3.1.6; https://pfind.org) (16) to identify the delta masses between the coding AAs and the detected residues. The peptide mass range was set to 600 Da - 6000 Da, the peptide length was 6 to 60,and the FDR for peptides was 1%. Modified peptides were then quantified from normalized peak area integration values with pQuant software (version: v202301; https://pfind.org). A catalog of all confidently identified peptides with a given delta mass cluster was constructed and normalized based on their total counts of the peptide or protein abundance.

For protein quantification analysis, raw MS data were searched against the human UniProt database (proteome ID: UP000005640, version: Release 2021_04) containing 20,350 unique sequences using Proteome Discoverer (version 2.1; https://www.uniprot.org/proteomes/UP000005640). Proteome Discoverer parameters were set to ±10 ppm precursor ion mass tolerance (instrument-specific for Orbitrap) and ±0.02 Da fragment ion mass tolerance. Acceptance criteria included FDR<1% (peptide level) for peptide-spectrum matches. A minimum of 1 unique peptide for a given protein was required in order to consider it as valid. Relative protein abundances were derived from normalized peak area integration values, coupled with retention time alignment and data normalization. MS1-based label-free quantification intensities of the protein groups were extracted from the Proteome Discoverer result files to represent the final expression of a particular protein across samples. All statistical tests and calculations were performed using Perseus within the Proteome Discoverer environment. For comparison between samples, the normalized iBAQ intensities were log2-transformed and the protein groups were filtered to have at least 70% valid values.

## Quantification and Statistical Analysis

### Data Normalization

Quantitative proteomic or ncAAs-omic profiles were z-scored or log2 transformed if necessary. Density maps of the normalized proteins expression or ncAAs-modified protein levels in each sample show the expected unimodal distribution, and all samples pass quality control. After filtered, the proteomic or ncAAs-omic matrix used for subsequent analysis is shown in Supplemental Tables S2 and S4.

### Quantitative Expression Analysis

Expression patterns of proteins or ncAAs-modified protein levels were first assessed using Mfuzz (19). The filtered and normalizated expression matrix was used for differential expression analysis. Algorithms of generalized linear models are used to evaluate molecular expression levels. Contrasted with healthy population, Log2 Foldchange (Log2 FC) of proteins or ncAA-sites in COVID-19 patients and recovered patients was respectively calculated. Bilateral ANOVA tests were performed for each comparison group and *p* values were corrected with benjamini-Hochberg algorithm (Adj. *p* value), ensuring adequate statistical significance for differential protein quantification. Adj. *p* value < 0.05 and |FC|>1.5 were set as the thresholds for selecting differential expression proteins or ncAA-sites. Differentially expressed proteins and ncAA-sites were subclustered with Hierarchical Clustering method. Venn diagram, heatmap, and network visualization were performed with vennDiagram, pheatmap, ggplot2 package, and Cytoscape V.3.5.129 software (https://cytoscape.org).

### Pathway Enrichment Analysis

Gene Set Variation Analysis (GSVA) (20) evaluated the performance of the Kyoto Encyclopedia of Genes and Genomes (KEGG) pathway gene sets in each sample. The human KEGG pathway gene sets were downloaded from KEGG website (https://www.kegg.jp/) and were constructed the GMT file to perform Gene Set Variation Analysis. Additionally, annotation of differentially expressed proteins or ncAAs-modified sites (DE-ncAA-sites) were performed in Metascape website (https://metascape.org/). Ingenuine pathway analysis and right-tail Fisher's test were used to identify the most significant pathways.

### Partial Least Squares Discriminant Analysis

The expression matrix of complement and coagulation cascades-related ncAAs-modified sites was extracted form ncAAs-omic profile and subsequently analyzed with Partial Least Squares Discriminant Analysis to evaluate the significant complement-related ncAAs-modified sites in COVID-19 patients and convalescents. 10 repeats of 5-fold cross-validation evaluated the classification performance of different principal component models, and the model with the highest accuracy was selected for further analysis.

### Identify Representative Features by Machine-Learning Strategy

To identify serum representative molecules of COVID-19 patients and convalescents, we first divided ncAAs-omic into different groups, including (1): For characterizing serum markers in COVID-19 patients, COVID-19 patients are positive group and healthy people are negative group (2); for characterizing serum markers in COVID-19 convalescents, COVID-19 convalescents are positive group and healthy people are negative group. The training cohort, sampled randomly and proportionally from the total 412 individuals at a proportion of 75%, was subjected a machine-learning strategy, named as RFI19. RFI19 consists of three steps: important features selection, candidate features selection, final features determination. In important features selection step, by filtering uncompetitive features with Boruta algorithm with parameters as pValue = 0.01, maxRuns = 100, we select serial important features for subsequently modeling. In candidate features selection step, we randomly select 5 features to generate combination, and prepare 1000 candidate combinations. In final features determination step, we randomly select 3 features to generate combination, and prepare 1000 candidate combinations. We used the candidate features to train model and evaluated the model performance with nested cross-validation (five outer folds + three inner folds). The least absolute shrinkage and selection operator (L1 regularization) penalty and ridge regression (L2 regularization) (α = 1 for least absolute shrinkage and selection operator, α = 0 for Ridge, grid search for λ (lambda) with 100 values (1e-4 to 1e2, log-scaled)) penalty to optimize the weights of candidate features. To simplify the features, if the AUC value of five-fold cross-validation increase, one feature is randomly removed until the AUC value is no longer increased. The optimal features combination model with the maximum AUC value is determined. The classification performance of the selected model was evaluated with five repeats of 10-fold cross-validation, utilizing the testing cohort which comprised of the remaining samples from the total of 412 individuals. We calculate true positives, true negatives, false positives, and false negatives. Then, sensitivity, specificity, accuracy and Kappa coefficient were calculated based on confusion matrix to evaluate the accuracy of RFI19.

## Results

### Widespread Non-Coded Amino Acids Identified as Molecular Imprints in the Proteome of COVID-19 Convalescent Serum

**Fig. 1:**
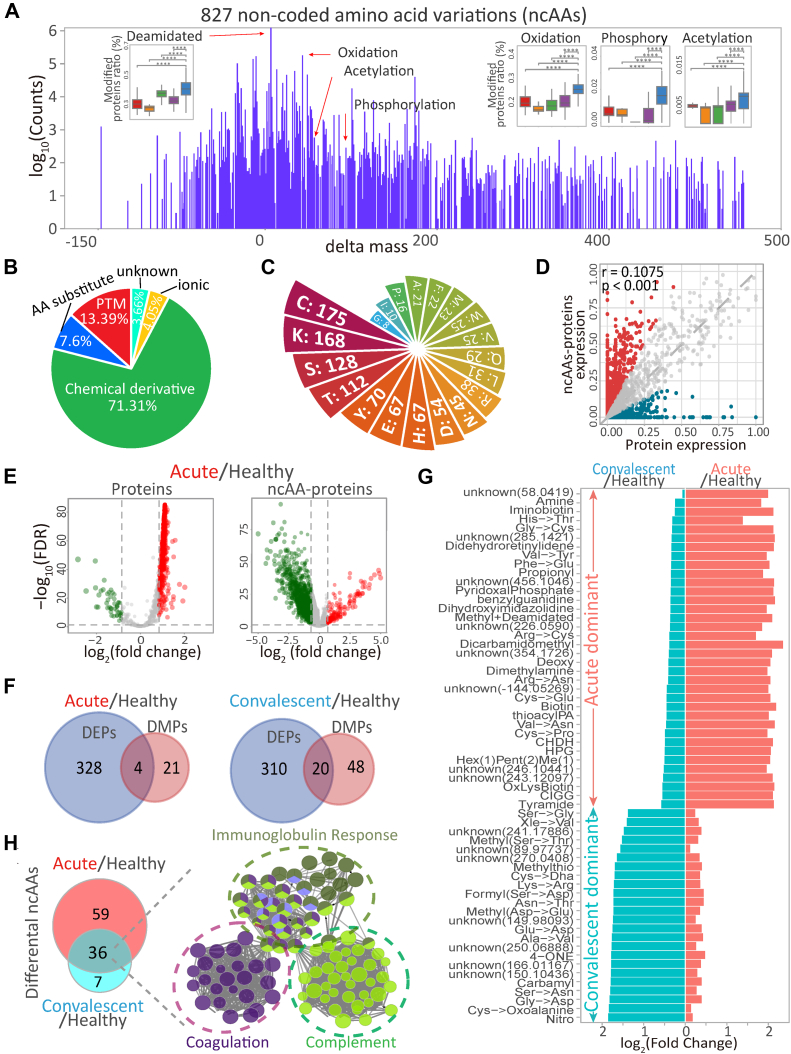
**Overview of serum ncAA-omic profile.***A,* delta masses identified from the human serum proteome dataset revealed a complex modification landscape. Boxplot showing modified proteins proportion in samples from 412 COVID-19 patients, convalescents and healthy populations. *B,* the distribution of peptide counts of different properties modifications. *C,* different AAs cluster with different number of modifications. *D,* correlation between all modified proteins abundance and related protein expression levels. *E,* volcano Plot showing differential expression proteins (*left*) and ncAAs-modified proteins (*right*) in acute patients, contrast to healthy populations. *F,* overlap of differental expression proteins (|fold change| > 2, FDR <0.05) and differental modified proteins (|fold change| >1.5, FDR <0.05) from the acute and recovery stages of COVID-19 patients, contrast to healthy populations. *G,* the representative modifications differentially expressed at the acute and recovery stages of COVID-19, contrasted to healthy populations. *H, left*: Overlap of differentially expression modifications (|fold change| > 1.5, FDR <0.05) at the acute and recovery stages of COVID-19. *Right*: Functional annotation of proteins modified with overlapped differentially expression modifications. AA, amino acid; FDR, false discovery rate; ncAA, non-coded amino acid.

Clinical indexes of 412 patients’ blood work demonstrated the abnormalities of several indicators such as the high levels of albumin, sodium, and potassium in the COVID-19 convalescent serum up to 12 months post infection **(**Supplemental Table S1, Supplemental Fig. S1*B***)**. To identify the molecular imprints in the serum proteins relevant with sequelae, an approach for global protein modifications was applied following an open-restricted search workflow (Supplemental Fig. S1*A*). Briefly, the shotgun proteomics was used to determine the accurate mass of the peptides (MS) and fragmented ions (MS/MS) of trypsin-digested serum proteins. The MS/MS profiles were matched with the non-redundant protein database, which identified 1685 serum proteins from the acute phase and convalescent patients (Supplemental Table S2). The unmatched peptide profiles were searched to identify the mass differences (delta masses) between the coding AAs and the detected residues using an open search algorithm (Supplemental Table S3) (17). The delta masses were clustered with Gaussian regression, filtered with the known modification databases (21), and used as a pre-selected delta mass list for a restricted search (16, 17) to generate the final list of non-coded amino acids and derivatives (Supplemental Table S4), termed ncAAs. Following this workflow, 827 different ncAAs were identified from the patient’s serum (Fig. 1*A*, Supplemental Table S4), theoretically representing structural variations of proteins by AA substitutions, post-translational modifications (PTMs), and unknown AA derivatives. These ncAAs were spreading over 29,814 protein sites, suggesting widespread AA polymorphisms or modified proteins on top of the proteomic expression. Accounting for the incidents of ncAAs, 207,345 (7.6%) matched AA substitutions, 365,383 (13.4%) were known PTMs, and 1,946,090 (71.3%) were annotated as chemical derivatives (Fig. 1*B*) (21), demonstrating widespread protein sequence and structural variations. The frequencies of PTMs and chemical derivatives reflected the reactivity of each AA in proteins following the order of Cys > Lys > Ser from the top, and Gly < Ile < Pro < Ala from the bottom (Fig. 1*C*). Notably, the ncAA occurrence was poorly correlated with protein expression (r = 0.107, *p* = 0.001) (Fig. 1*D*, Supplemental Fig. S1, *C* and *D*). Thus, widespread occurrence of ncAAs suggested an independent omics at the structural level on top of proteomic expression, stratifying in COVID-19 patients, convalescent and healthy populations. In addition, the differentially modified proteins by ncAA and differentially expressed proteins were independently regulated in the acute and convalescent phases (Fig. 1, *E* and *F*, Supplemental Fig. S2, Supplemental Table S4). Common PTMs and chemical derivatives such as methylation, nitration, formylation and many unknown ncAAs were found predominately in the acute phase of infection (Fig. 1*G*). In contrary, propionylation, pyridoxal-phosphorylation and unknown modifications were predominately found in the convalescent serum. Markedly, many of the ncAAs and ncAA-modified proteins were persistently present in convalescences even 12 months after recovery, and these differentially modified proteins were predominantly involved in the immune response, complement activation and fibrinolysis coagulation in the circulating proteins (Fig. 1*H*, Supplemental Fig. S1*E*).

### Widespread ncAAs Associated with Immune Responses in COVID-19 Convalescent Serum

**Fig. 2:**
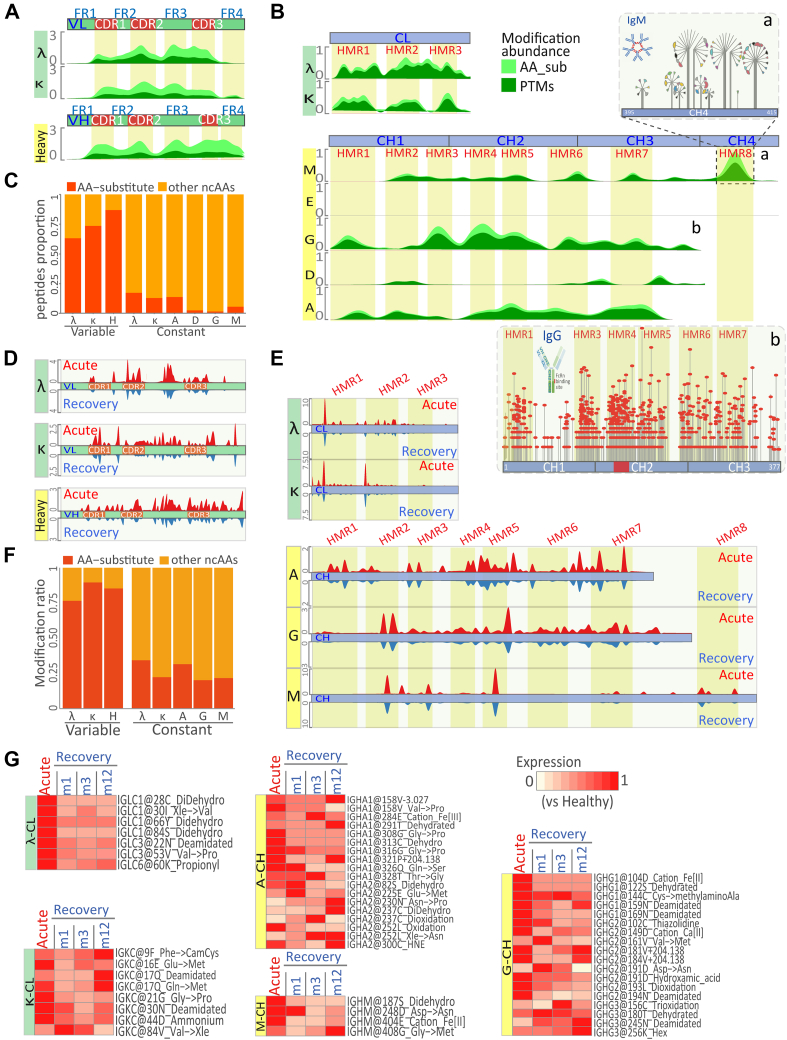
**The sustained ncAA imprints in serum immunoglobulin across COVID-19 course.***A and B,* the schematic showing the diversity and abundance of AAs and ncAAs along with the immunoglobulin variable (*A*) and constant (*B*) chains. a:The ncAAs distribution at modified hotspot (HMR8) in C-terminal of lgM heavy chain. b:The ncAAs distribution at modified hotspot along with lgG heavy chain. *C,* the ratio of amino-acids substitutes (AA-substitute) or other ncAAs along with the immunoglobulin chains. *D and E*, the ncAAs expression pattern along with the light (*D*) or heavy (*E*) immunoglobulin chains in acute (*red*) or recovery (*blue*) stages of COVID-19 patients against health populations. *F,* the ratio of sustained differentially expressed amino-acids substitutes (AA-substitute) or other ncAAs along with the immunoglobulin chains. *G,* the expression pattern of representative sustained ncAAs imprints in immunoglobulin constant chains across COVID-19 course. ncAA, non-coded amino acid.

To explore the molecular imprints in immunoglobulin responses, ncAA-containing immunoglobulins were retrieved from the patients and convalescents. A total of 7122 ncAA-modified sites were found within the variable regions of λ, κ, and heavy chains, and the constant regions of IgM, IgG, IgD and IgA (Supplemental Table S5). Among them, AA substitutions, PTMs and undefined AA chemical derivatives were also detected, suggesting ncAA-modified proteins played important roles in immunoglobulin-mediated host responses to infection. Notably, a great portion of AA-substitutions were found within immunoglobulins, particularly for tyrosine and serine (Supplemental Fig. S3*A*). For immunoglobulin, three hyper ncAA-modified regions (HMRs) in the variable regions of λ, κ and heavy chains were identified that matched the complementarity-determining regions known as CDR1, CDR2, and CDR3 (Fig. 2*A*, Supplemental Fig. S3*B*) (22). Notably, the most ncAAs within HMRs (>75%) were AA-substitutions (Fig. 2*C*) in agreement with antibody somatic (V(D)J) recombination (23). Two previously undefined HMRs were found in the framework regions between CDRs (Fig. 2*A*): the framework 3 between CDR2 and CDR3 in the λ, κ and heavy chains, and the framework 4 near CDR3 predominantly in the heavy chain. Indeed, a significant fraction of PTMs and chemical modifications were detected in the variable regions of the light and heavy chains (Fig. 2, *A* and *C*), suggesting that, on top of somatic (V(D)J) recombination (23), ncAA-modifications provided an additional level of regulation for antigen-antibody interaction in immunoglobulin responses.

Several hotspots (HMR1-8) were also detected in the constant regions (Fig. 2*B*, Supplemental Fig. S3*B*), which reasonably generated structural diversities to provide a variety of regulatory variants for downstream immune responses. Three HMRs (HMR1-3) were found within the κ and λ constant light chain (CL), although certain ncAAs were also seen in between the HMRs in the λ chain (Fig. 2*B*, upper panel). Within the constant heavy (CH) chain, however, the hotspots HMR1-8 were clustered differently in the IgM, IgG, and IgA chains, except IgD and IgE (Fig. 2*B*, lower panel). HMR1-2 was found in the CH1 region, HMR4-5 in the CH2 region, HMR7 in the CH3 region, and HMR3/6 near the transitional regions between CH1, CH2 and CH3. Notably, IgG and IgA were extensively modified across HMR1-7, suggesting the importance of ncAA-modifications in regulating immunoglobulin-directed immune activation. In support, the hotspot HMR3-5 was overlapped with the complement and Fc receptor binding sites (22) (Fig. 2*B*, Supplemental Fig. S3*B*). The hotspot HMR8 was only found in the CH4 region important for IgM pentamer formation (24) (Fig. 2*B*, upright). In contrary to the dominant AA-substitutions in the variable region, ncAAs in the constant regions were predominantly PTMs and AA derivatives (>70%) (Fig. 2, *B* and *C*), which were reasoned to generate a variety of structural diversities important for the regulation of downstream immune responses.

Markedly, many ncAAs were induced in the acute phase of infection and some sustained in the convalescents (Supplemental Table S5), termed COVID-19-associated ncAAs. For the variable region, most COVID-19-associated AA-substitutions in the λ and κ light chains (VL) appeared in the acute phase and sustained in the convalescences even 12 months after the recovery (Fig. 2, *D* and *F*, Supplemental Fig. S3*C*). In contrary, the induced AA-substitutions in the variable heavy chain apparently returned to normal and disappeared during convalescences. For the constant region, the COVID-19-associated ncAAs were mostly PTMs and chemical derivatives, some of which disappeared but most sustained in the convalescences (Fig. 2, *E* and *F*, Supplemental Fig. S3*C*). Notably, these COVID-19-associated ncAAs were identified in the hotspots (HMR3-5) important for the complement and the Fc receptor binding sites, suggesting potential effects on the downstream of immune responses associated with COVID-19. A few typical COVID-19-associated AA-substitutions were found in the hotspot HMR4-7 of IgA (IGHA1@308G > P, 316G > P, 326Q > S, and 328T > G), and in the hotspot HMR8 of IgM (IGHM@408G > M). Typical COVID-19-associated PTMs and chemical derivatives included the hotspot HMR3-6 of IgG2 (IGHG2@102C + Thiozlidine, 149D + Ca^2+^, 181V + 204.138, 184V + 204.138, 191D + Hydroxamic acid, 192L + dixodation and 194N-NH3), IgG3 (IGHG3@245N-NH3 and 256K + Hex), and IgM (IGHM@404E + Fe^2+^) (Fig. 2*G*). Thus, COVID-19-associated ncAAs as molecular imprints were reasoned to affect antibody-antigen recognition complement activation and Fc receptor interaction in the acute phase and COVID-19 convalescents.

### Widespread ncAAs Associated with Complement Activation and Coagulation Regulation in COVID-19 Convalescent Serum

**Fig. 3:**
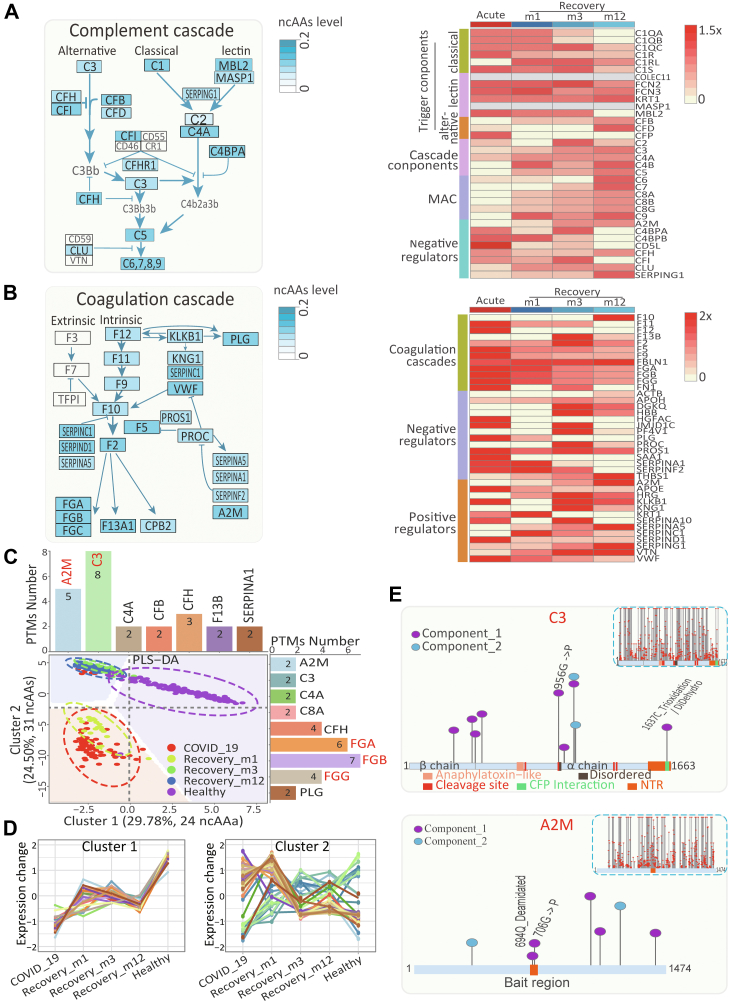
**The sustained ncAAs imprints of serum complement and coagulation components across COVID-19 course.***A, left*: The distribution and abundance of ncAAs in complement components. *Right*: The abundance of ncAAs in complement across the acute and recovery stages of COVID-19 patients, contrasted to healthy populations. *B,* left: The distribution and abundance of ncAAs in coagulation components. *right*: The abundance of ncAAs in coagulation cascades across the acute and recovery stages of COVID-19 patients, contrasted to healthy populations. *C,* partial least squares discriminant analysis of the ncAAs enriched in complement and coagulation cascades. *Histogram* indicates the proteinic localization of ncAAs in component 1 (*up*) and 2 (*right*). *D,* dynamic expression patterns of normalized ncAAs in component 1 (*left*) and 2 (*right*) across COVID-19 course. *E,schematic* showing the representative ncAAs enriched in component 1 and 2 or global ncAAs (*Upright*) in C3 (*up*) or A2M (*down*). ncAA, non-coded amino acid.

To explore the molecular imprints associated with complement and coagulation, ncAAs-modified complement and coagulation proteins were retrieved from the patients and convalescents (Supplemental Tables S6 and S7). Among them, 61 typical COVID-19-induced and ncAA-containing complement components were closely associated with the classical, alternative, and lectin pathways in the acute phase of infection (Fig. 3*A*), suggesting that these ncAAs were involved in complement activation commonly associated with the inflammatory responses during COVID-19. Comparing with healthy population, these ncAAs on the triggering components such as C1QA, C1QB, C3, CFB, CFP and MBL2 in all three activation pathways were significantly induced during infection and eventually returned to normal after recovery (Fig. 3*A*). However, there were several ncAAs sustained to be upregulated in the convalescents even 12 months after the recovery, including the triggering component C1QC, C1R, C1RL and C1S in the classical pathway, KRT1, FCN2 and FCN3 in the lectin pathway, and CFB and CFD in the alternative pathway. Similarly for the amplificatory cascade and membrane attack complexes, ncAAs on C3, C5, C7, C8A, C8B and C9 were recovered; however, the induced-ncAAs on C4A, C2, C6 and C8G still sustained. Notably, the inductionS of ncAAs on most negative regulators were recovered after the acute stage, except that COVID-9-induced ncAAs on CLU sustained in the convalescents 12 months after recovery. In contrary, ncAAs in the coagulation pathway were induced in COVID-19 and mostly recovered in the convalescents (Fig. 3*B*). Obviously, only ncAAs in the endogenous coagulation factors were regulated across COVID-19.

To characterize the expression pattern of ncAAs across different stages of COVID-19 course, ncAAs from the complement and coagulation components were clustered by partial least squares discriminant analysis (Fig. 3*C*, Supplemental Fig. S4). Two clusters of ncAAs, 24 in cluster 1 (29.78% heterogeneity) and 31 in cluster 2 (24.5% heterogeneity), were found to efficiently distinguish the patients in the acute phase, recovering stages and the healthy population. The ncAAs in cluster 1 (A2M, C3, C4A, CFB, CFH, F13 B and SERPINA1) were able to separate the healthy population from the patients in the acute and recovery stages. Notably, ncAAs in cluster 1 were mostly the complement cascade components that downregulated and sustained even after 12-months recovery (Fig. 3*D*, left). The ncAAs in cluster 2, primarily enriched in the complement pathway (A2M, C3, C4A, CFH, etc) and coagulation pathway (FGA, FGB, FGG ect), were found to efficiently distinguish the patients in the acute phase and convalescents. Markedly in cluster 2, the ncAAs in the coagulation pathway returned to normal in convalescences; however, the ncAAs in the complement pathway didn’t (Fig. 3*D*, right). Therefore, it suggested that the induced-ncAAs in complement components were sustained and served as molecular imprints for COVID-19 convalescents.

In support, most COVID-19-induced ncAAs occurred near the protein sites crucial for complement activation and coagulation regulation (Fig. 3*E*). For the complement cascade component C3, the hotspots on C3 and A2M, C3-related modifications enriched in Partial Least Squares Discriminant Analysiswere uniformly distributed in the α and β chains, especially in C3 cleavage sites, anaphylatoxin-like domain and CFP interaction regions. As inflammatory regulator and the antigen conducting the hexamerization of IgG(25), modifications of 694C and 706C in A2M bait region are obviously enrich in component1, contributing to indicate that SARS-CoV-2 infection causes the sustained dysregulation in complement activity.

### COVID-19-Associated ncAAs as the Potential Targets for COVID-19 Sequalae

**Fig. 4:**
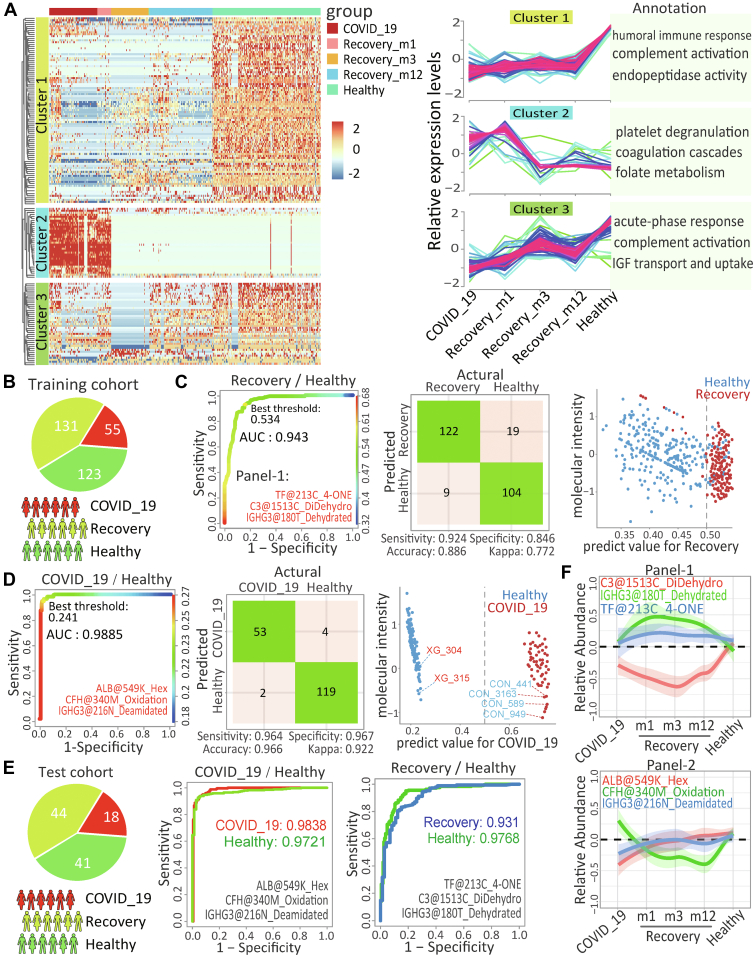
**Characteristic the representative ncAAs in acute and recovery stages of COVID-19 patients.***A, left*: Heatmap showing the expression pattern of differentially ncAAs at the acute and recovery stages of COVID-19, contrasting to healthy populations (Cutoff: |fold change| > 1.5, FDR <0.05). *Hierarchical clustering* indicated ncAAs were significantly grouped. *Right*: Dynamic expression pattern and functional annotation of marked clusters. *B,* Overview of serum samples collection for training the machine learning process, including 55 acute and 131 recovery patients and 123 healthy populations. *C,* ROC curve (*left*), confusion matrix (*middle*), performance (*right*) of indicated ncAAs model for classing COVID-19 convalescents and healthy populations in the train cohort. *D,* ROC curve (*left*), confusion matrix (*middle*), performance (*right*) of indicated ncAAs model for classing acute COVID-19 patients and healthy populations in the train cohort. *E,* overview of serum samples collection for testing the machine learning process, including 18 acute and 44 recovery patients and 41 healthy populations (*left*). ROC curve of indicated ncAAs model for classing acute COVID-19 patients or COVID-19 convalescents from healthy populations in the test cohort. *F,* parallel Reaction Monitoring analysis quantifying serum levels of characteristic non-canonical amino acid (ncAA)-modified peptides in healthy controls (n = 40), acute COVID-19 patients (n = 40), and convalescents (n = 40). Values are expressed as mean ± SEM. Corresponding representative ion chromatograms and annotated MS/MS spectra for verified peptides are shown in Supplemental Figure S7. ROC, receiver operating characteristic; ncAA, non-coded amino acid.

To further explore the ncAAs as the potential targets in association with different stages of SARS-CoV-2 infection, 29,814 ncAA-sites over 1685 serum proteins were clustered across the COVID-19 course and convalescences, which supported that some of the dysregulated ncAAs (group 1) were sustained in the convalescents (Supplemental Fig. S5, *A* and *B*). Among them, the top 95 differentially expressed ncAAs in acute COVID-19 stage (|FC|>1.5, FDR<0.05) were retrieved (Supplemental Fig. S5*C*) and sub-clustered (Fig. 4*A*). Typically, ncAAs in cluster 2 were exclusively upregulated in the acute phase of SARS-CoV-2 infection but returned to normal after recovery within 1 month. These ncAAs were mainly involved in the coagulation pathway, suggesting that the upregulated ncAAs participated the acute response of coagulation and did not leave molecular imprints in the convalescents. In contrary, ncAAs in cluster 1 were downregulated in acute phase and the downregulations sustained in convalescents 12 months after recovery. These ncAAs were apparently associated with complement components and immune responses, suggesting that the downregulated ncAAs remained in the convalescents and were responsible for long-term sequelae of COVID-19. Briefly, 19 of 95 COVID-19-assocated ncAAs appeared in all stages of infection (Supplemental Fig. S5*C*), including protein phosphorylation and deamination in the complement and coagulation components such as FGA@22S + phosphate and C1S@344N + 0.984.

To identify the molecular imprints that could predict sustained dysregulation or the potential risk of sequalae for the recovered COVID-19 patients, ncAAs from a cohort of 309 COVID-19 patients, convalescents and healthy people were classified by a machine learning workflow (Fig. 4*B*, Supplemental Fig. S6*A***)**. By comparing the convalescents with the healthy population, a panel of 3 ncAAs (panel-1) including TF@213C_4-ONE, C3@1513C_DiDehydro, and IGHG3@180T_Dehydrated efficiently identified the fully recovered patients that would have undetectable molecular imprints (AUC=0.943), which was further conformed by confusion matrices (Fig. 4*C*) and unrecovered expression pattern (Fig. 4*F*). The reliability of prediction was validated by the second cohort of 102 COVID-19 patients, convalescents, and healthy populations (Fig. 4*E*). Thus, the panel-1 efficiently separated the convalescents into partially recovered patients (AUC = 0.931) and fully recovered patients (AUC=0.977) with sufficient accuracy (Fig. 4*E*).

Similarly, panels of 3 ncAAs (panel-2) including IGHG3@216N_Deamidated, CFH@340M_Oxidation, ALB@549K_Hex were obtained to determine the prognosis for the acute COVID-19 patients with sufficient accuracy to separate the acute patients from the healthy people (AUC=0.989, Fig. 4*D*), for their significant expression switch in acute stage of COVID-19 (Fig. 4*F*), which was confirmed by the second cohort (Fig. 4*E*). Thus, the combination of the panel-2 could be used to predict whether the acute COVID-19 patients would fully recover or develop molecular imprints that would not disappear in the convalescent stages.

### COVID-19-Associated ncAAs Differ from COVID-19-Associated Proteins Alternation

In parallel with Shen's serum proteomic profiles from COVID-19 acute patients, SARS-CoV-2 infection induced abnormally expressed proteins in acute phase were mainly associated with complement, coagulation, lipid metabolism, and cell death (Supplemental Figs. S7 and S8). The differently expressed proteins primarily involved in the initial triggering of complement MBL pathways, such as MBL, MASP1/2, FCN2, C4B (Supplemental Fig. S7*C*). This is different from differential expressed ncAAs in acute patients, which were mainly involved in proteins enriched in initial triggering of complement alternative pathway and complement cascade components, such as A2M, C3, C4A, CFB, CFH, etc (Supplemental Figs. S5*D*, S9).

Finally, five proteins combinations (CLU, SERPINA1, APOA1, THBS1, CPN1), to distinguish COVID-19 patients, convalescents and healthy individuals, were further assessed by machine learning workflow. Based on proteomic profiles from cohort 1 and cohort 2, the 5-fold cross-validation AUC values were calculated as 0.9838, 0.9265 and 0.9721(Supplemental Fig. S10*A*). Both the confusion matrix and NMDS results showed that different samples can be correctly classified with high accuracy (Supplemental Fig. S10, *B*–*D*). Those results indicated the difference of serum representative molecular alternation between ncAAs-modified proteins and protein expression levels, no matter in COVID-19 patients or convalescents.

## Discussion

We have applied a restricted open-search workflow to identify possible molecular imprints left on serum proteins after SARS-CoV-2 infection, measured by the mass differences between the coding AAs and actual residues termed non‑coded amino acids (ncAAs). The detection of widespread ncAAs indicates coronavirus-caused structural and functional alterations on top of human proteome. Our data unraveled a total of 827 different ncAAs over 29,814 sites on serum proteins, representing a wide spectrum of structural and functional variations of proteins (Fig. 1). As the molecular imprints of coronaviral infection, these ncAAs were differentially regulated in COVID-19 patients and convalescents, many of which sustained even 1 year after recovery. Consistently, the dysregulated and sustained ncAAs in COVID-19 convalescents were functionally correlated with the pathways relevant to immune responses, complement activation, and coagulation, suggesting the biological impacts of the molecular imprints. In support, chronic inflammation is reported in association with a range of health problems after coronaviral infection (26). Typical serological features include dysfunction of immune system such as complement-driven activation of CD16^+^T cells and increased inflammatory capacity (27, 28) and proteomic studies support the dysregulation of host immune response and coagulation pathways in COVID-19 convalescents (29, 30, 31). In brief, because the occurrence of ncAAs is independent of ncAA-modified protein expression, the omics of ncAA unveils a different dimension of network at the levels of protein structure and function important for the pathogenesis of COVID-19 sequelae.

The ncAAs typically AA-substitutions within the CDR and framework regions of immunoglobulin are agreed with the somatic recombination of immunoglobulin at the DNA level (23). Notably, a significant fraction of PTMs and chemical modifications is detected in the variable regions and many of the alterations sustained in the convalescents (Fig. 2). It unveils that, on top of somatic mutations, post-translational protein modifications are important factors to regulate the specific recognition between the newly emerged antigens and antibodies. Our workflow can be applied to systematically identify and monitor virus-induced specific antibodies in COVID-19 convalescents. Markedly, more ncAAs are found in the constant regions, particularly near the motifs important for immune receptor recognitions, including the complement-binding sites of IgG and the pentamer-forming sites of IgM. As many of the ncAAs within FcRn are induced in the acute phase and sustained in the convalescents even 12 months after recovery, persistent complement activation and immune responses are reasoned to last for a long period of time. In addition, extensively modified complement components are also observed in association with the trigger processing, the cascade initiating, and the membrane attacking (Fig. 3). Therefore, the omics of ncAAs represents a characteristic pattern of molecular imprints that are responsive for the persistent complement activation and immune dysregulation in COVID-19 convalescents. Indeed, it is shown that immunoglobulin-mediated cytotoxicity and complement hyper-activation are critical for the pathogenesis and the sustained damage of neural cell, gastrointestinal (GI) distress (28, 32, 33, 34). However, the biological significance of those ncAAs in CDR/HMR regions of immunoglobulin or functional domains of complements remains to be functional validated.

Notably, the widespread ncAAs on serum proteome represent a wide spectrum of AA variations including AA-substitutions, chemical derivatives and enzymatic modifications (35). In addition to directly affect immunoglobulin-mediated responses and complement-mediated attacking, the widespread ncAAs in proteins would generally alter the chemical properties and molecular functions, for instance, to change the immunogenicity and generate new antigens (34, 36, 37). The commonly induced modifications by coronaviral infection include deamination, oxidation, hydroxylation, dehydrogenation and sulfation. It is worthy to emphasize that deamidations of asparagine (N) and glutamine (Q) are among the top chemical modifications in the host cells, resulting in the addition of negative charges at the original positions (Fig. 2). Deamidation is a spontaneous and irreversible posttranslational modification that are shown to affect protein function and stability (38). In support, deamidation of N481 and N501 in the receptor binding motif of SARS-CoV-2 spike protein is reported to have an extended half-lives and enhanced immunogenicity (38, 39). The N501 mutation is the characteristic marker for the emerging SARS-CoV-2 variants of B.1.1.7 (UK), B.1.351 (South-Africa) and P.1 (Brazil) (40, 41, 42). In our study, deamidations of asparagine are induced in immunoglobulins and complement components in the acute patients, which are not reversed in the convalescents even 12 months after COVID-19 recovery. Therefore. deamination is a hallmark event of coronaviral infection and a persistent molecular imprint in COVID-19 convalescents.

In conclusion, we indicated COVID-19 convalescents are characterized by the sustained dysregulation of immunological ncAAs in immunoglobulin and complement, providing new insights into the molecular mechanisms of post-COVID-19 sequelae and potential interventions.

## Data Availability

The mass spectrometry raw data (.raw and search profiles) are deposited in iProX (https://www.iprox.cn/) with the Project ID IPX0013856000. Aggregated datasets are available upon reasonable request to the corresponding author (jhy@zzu.edu.cn).

## Supplemental Data

This article contains supplemental data.

## Conflict of Interest

The authors declare no competing interests.
