# Supplementary material for: Widespread Molecular Imprints in the Serum Proteome of COVID-19 Convalescents Uncovering Immune System Sequelae
Source: Mol Cell Proteomics. 2026 Feb 12;25(4):101525. doi: 10.1016/j.mcpro.2026.101525 (PMC13068859; doi:10.1016/j.mcpro.2026.101525)
Supplement: Supplemental Figures [file mmc10.docx]

**Supplementary Materials**

**Molecular Imprints on the Proteome of COVID-19 Convalescents Uncovering Sustained Multiple System Dysregulations**

Kun Liu^1#^, Zhigang Ren^1,2#^, Bowen Dong^1#^, Wenli Liu^2#^, Yuyuan Gao^1^, Li Zhang^1^, Jingyi Li^2^, Zhao Sun^3^, Hongyi Li^2^, Qian Zhao^1^, Xinchao Hu^1^, Jinfeng Chen^1^, Yuanyuan Wang^1^, Yang Yang^1^, Lei Zhang^1^, Xinli Xue^1^, Aiguo Xu^2^*, Zujiang Yu^2^*, Jing-Hua Yang^1^*

Lead Contact: Jing-Hua Yang, [jhy@zzu.edu.cn](mailto:jhy@zzu.edu.cn)

**Supplemental Tables:**

Supplemental Tab. S1. Baseline Characteristics for COVID-19 Patients,Convalescents, and Healthy Populations

Supplemental Tab. S2. Proteomic Profile for COVID-19 Patients,convalescents, and Healthy populations

Supplemental Tab. S3. Delta mass clusters by open search - Byonic

Supplemental Tab. S4. Atlas of ncAA protein sites by restrict search

Supplemental Tab. S5. ncAA-modified immunoglobulins

Supplemental Tab. S6. ncAA-modified complement proteins

Supplemental Tab. S7. ncAA-modified coagulation proteins

Supplemental Tab. S8. All quantified peptides informations

**Supplemental Figures**


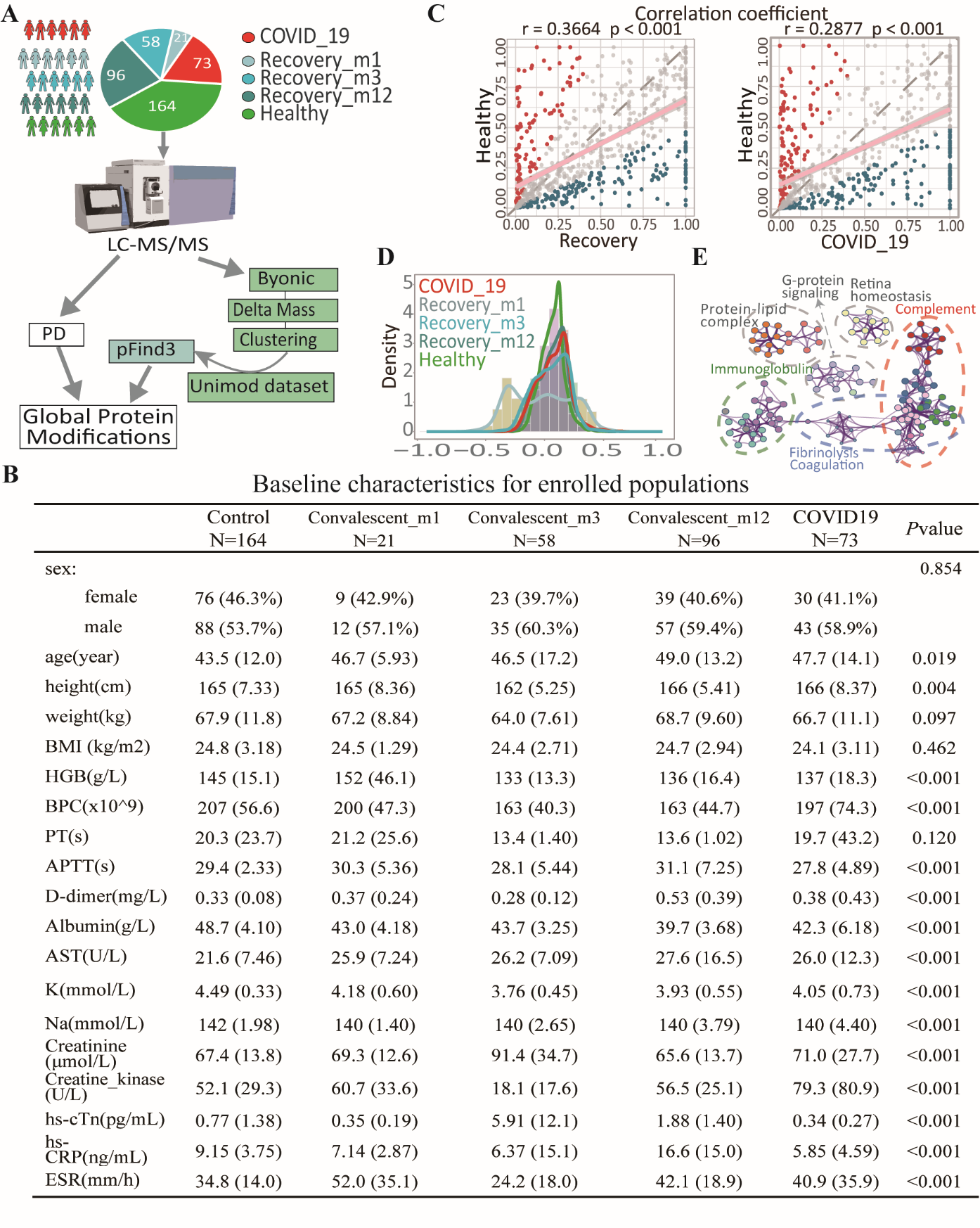


**Fig. S1.** Serum indicators expression pattern, workflow for global protein modification analysis, overview of serum ncAAs-omic profile.

**(A)** An approach for global protein modifications was applied following an open-restricted search workflow. Briefly, total serum proteins from acute COVID-19 patients, convalescents of 1, 3 and 12 months, and healthy populations are analyzed by the shotgun proteomics technology. The MS and MS/MS data are analyzed with matched with Protein Discovery (ver 2.4) for proteomics, and Byonic and pFind3 for unmatched amino acid residues. The delta masses from Byonic are clustered by Gaussian regression, annotated according to Unimod and combined with pFind3 to establish the atlas of global protein modifications.

**(B)** Baseline characteristics of the enrolled population and the comparisons between COVID-19 acute or convalescent patients and healthy populations. Clinical indexes of 412 patients’ blood work demonstrated the abnormalities of several indicators such as the high levels of albumin, sodium, and potassium in the COVID-19 convalescent plasma up to 12 months post infection.

**(C)** Correlation coefficient distribution between modified proteins abundance and related protein expression levels from the acute and recovery stages of COVID-19 patients and healthy populations.

**(D)** Correlation of modification expression pattern from the acute and recovery stages of COVID-19 patients and healthy populations.

**(E)** Functional annotation of all modified proteins.


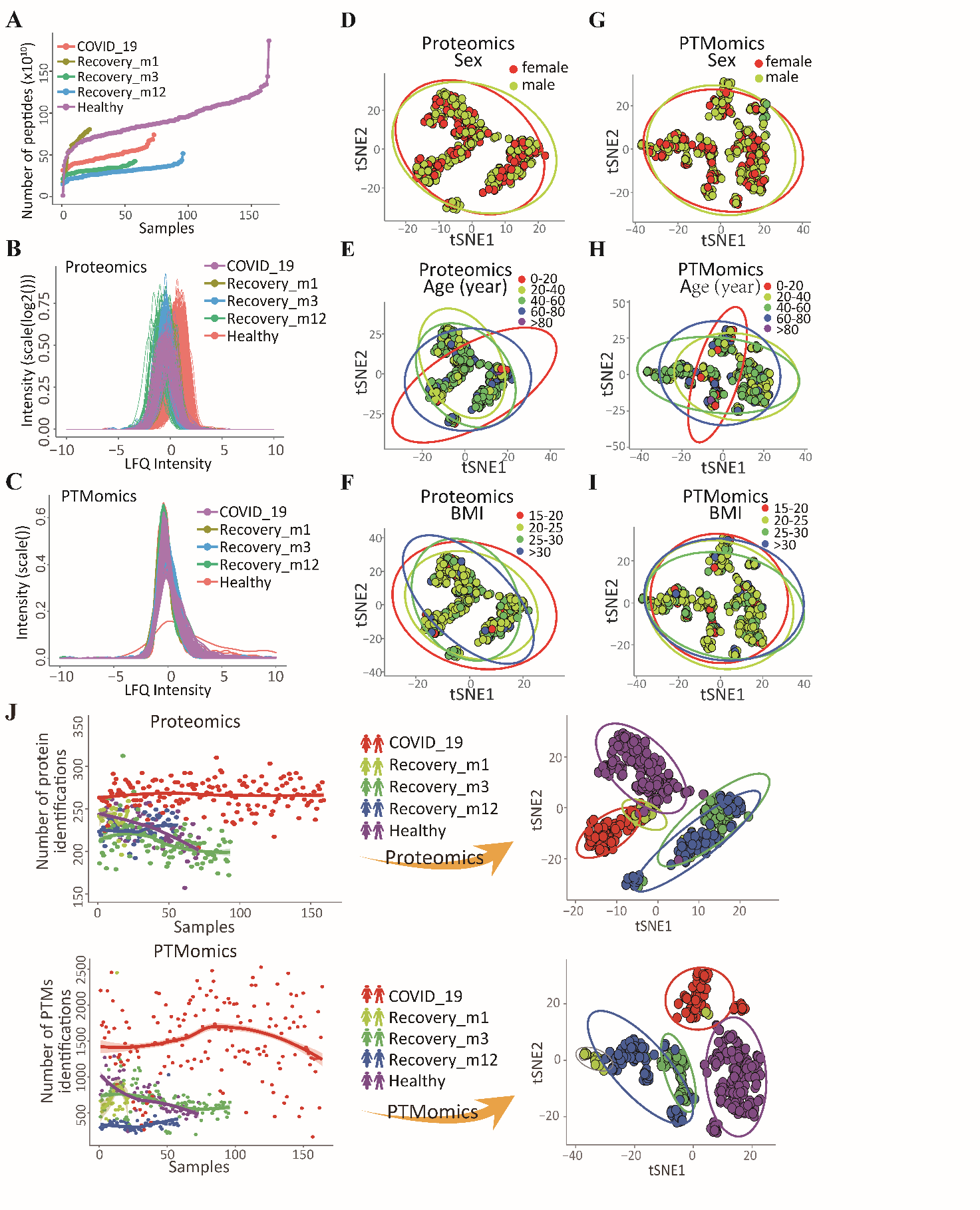


**Fig. S2.** The characteristics of serum proteomics and global ncAAs-omics from the acute and recovery stages of COVID-19 patients, and healthy populations.

**(A**) The statistics of mass spectrometry identified peptides.

**(B, C)** The normalized intensity distribution of proteomic **(B)** and ncAAs-omic **(C)** profile in groups. The intensity-based absolute quantification (iBAQ) value were log2-transformed or Z-scaled.

**(D-I)** Stochastic Neighbor Embedding analysis of the proteomic **(D, E, F)** and ncAAs-omic **(G, H, I)** Profiles based on sex, BMI(kg/m^2^) or age(years) of samples in the cohort.

**(J**) The distributions of serum quantitative proteomics and ncAAs by groups. Left, the dynamics of protein (up) or ncAAs (down) abundances; Right: Stochastic Neighbor Embedding analysis (tSNE) showed differences of proteomics (up) or ncAAs-omics (down) by groups.


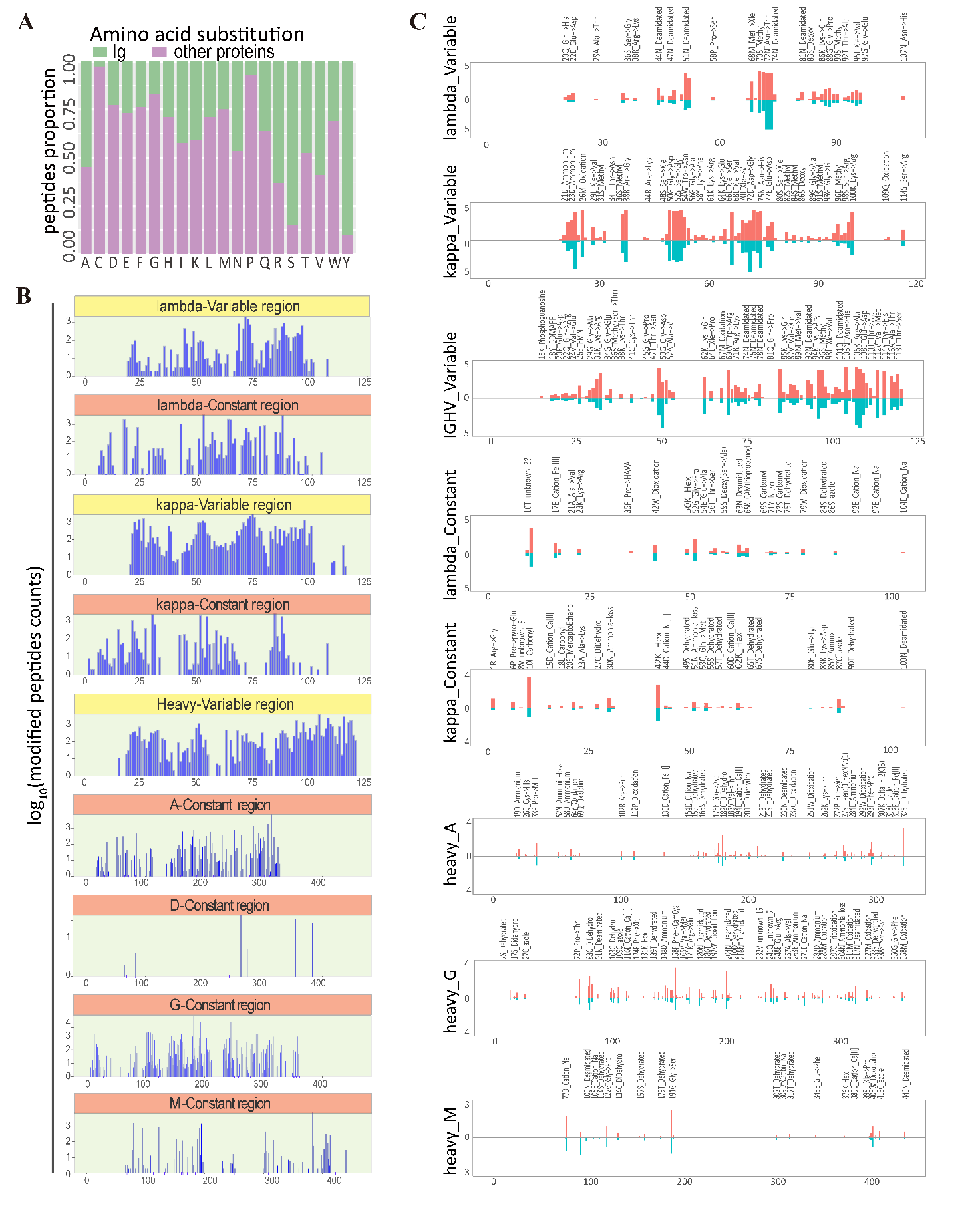


**Fig. S3.** The ncAAs imprints in serum immunoglobulin across COVID-19 course.

**(A)** The proportion of ncAAs-contained peptides counts summarized by amino acids in immunoglobulin, contrast to other proteins.

**(B)** The ncAAs modified peptides counts along with immunoglobulin chain.

**(C)** The sustained differentially expressed ncAAs (FDR<0.05) distribution along with immunoglobulin chain.


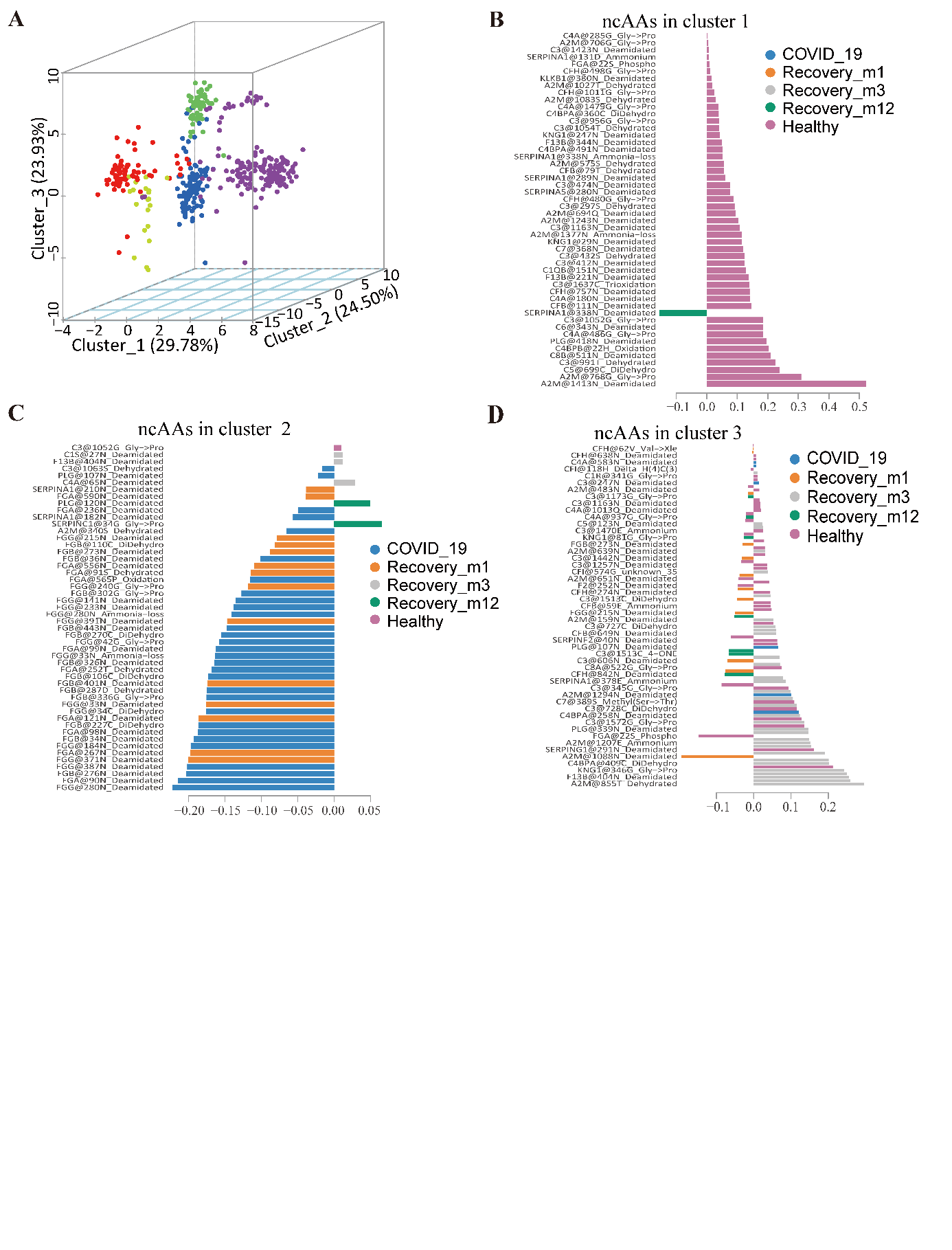


**Fig. S4.** The sustained dysregulation of serum complement and coagulation associated modified proteins.

**(A)** 3D scatterplot showing PLS-DA of the ncAAs modified peptides (ncAA-sites) enriched in complement and coagulation cascades pathway.

**(B, C, D)** Bar plot showing the ncAA-sites contributing to the component 1 **(B)**, 2 **(C)**, and 3 **(D)**.


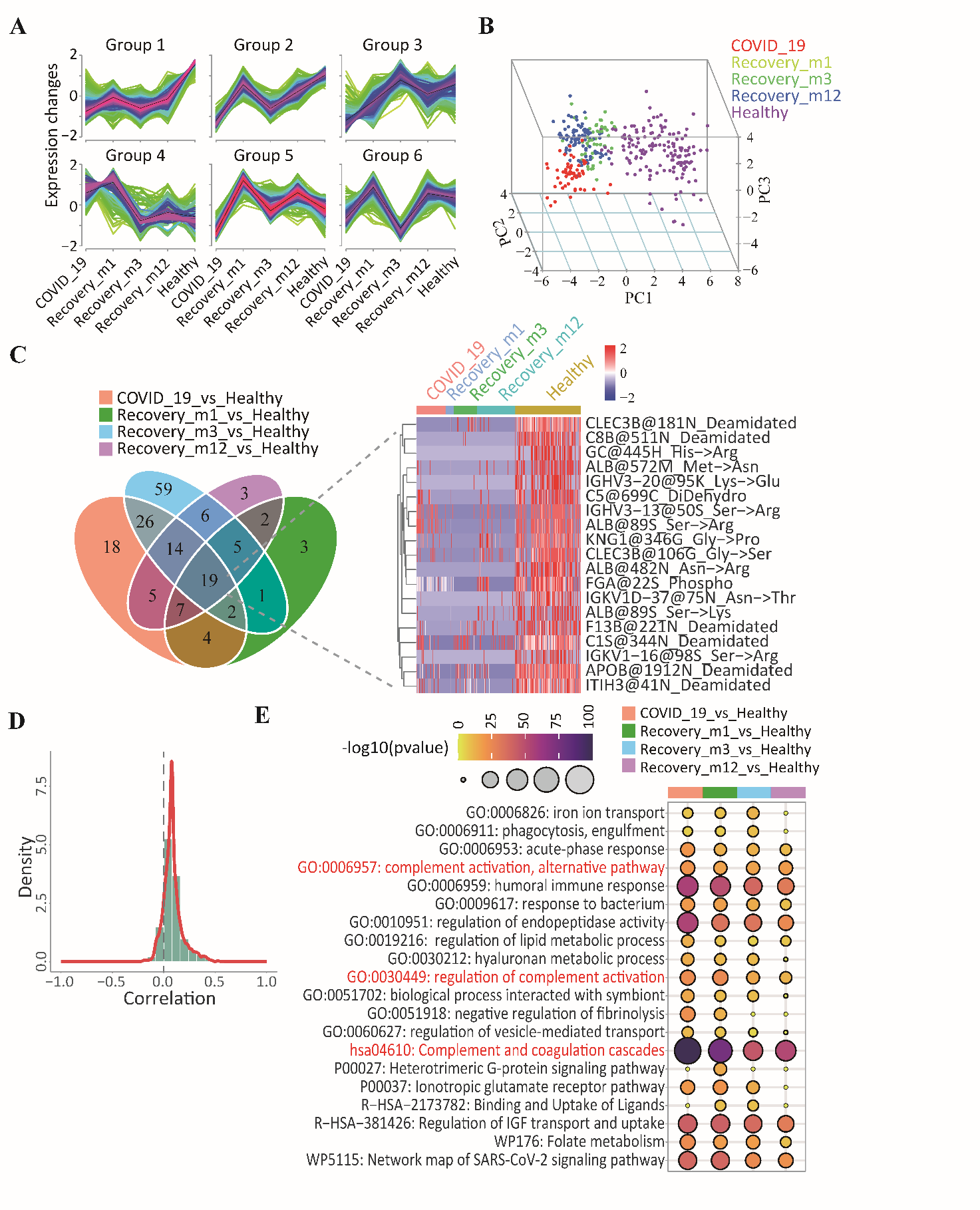


**Fig. S5.** Quantitative analysis of serum ncAAs across COVID-19 course.

**(A)** Expression pattern of all ncAAs from the acute and recovery stages of COVID-19 patients, and healthy populations.

**(B)** Principal component analysis (PCA) showed different expression pattern of ncAAs from the acute and recovery stages of COVID-19 patients and healthy populations.

**(C)** Left: Venn plot showing the differentially expressed ncAAs from the acute and recovery stages of COVID-19 against healthy populations. Right: Heatmap showing the expression pattern of shared differentially expressed ncAAs .

**(D)** Correlation between DMPs expression levels with their corresponding proteins expression levels.

**(E)** Functional annotation of differential expressed ncAAs in each contrasts.


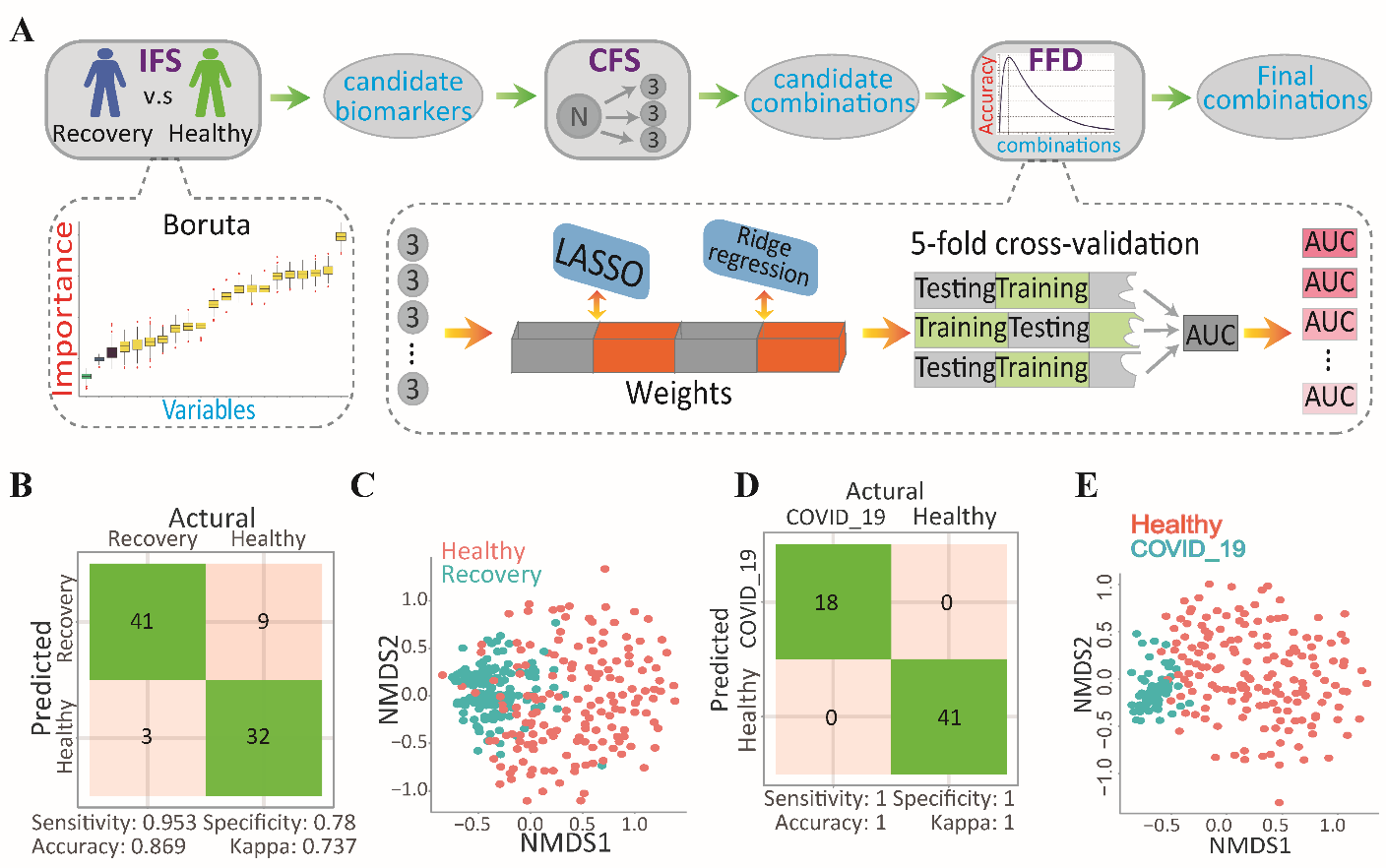


**Fig. S6.** Characteristic the representative ncAAs by machine-learning Strategy.

**(A)** Schematic machine learning processes for identifying biomarker combinations, including IFS (Important Features Selection), CFS (Candidate Features Selection) and FFD (Final Features Determination).

**(B, C)** Confusion matrix **(B)**, non-metric multidimensional scaling (NMDS) assay **(C)** of indicated ncAAs (in Fig. 4C) for classing COVID-19 convalescents and healthy populations in the test cohort.

**(D, E)** Confusion matrix **(E)** and NMDS assay **(F)** of indicated ncAAs (in Fig. 4D) for classing acute COVID-19 patients and healthy populations in the test cohort.


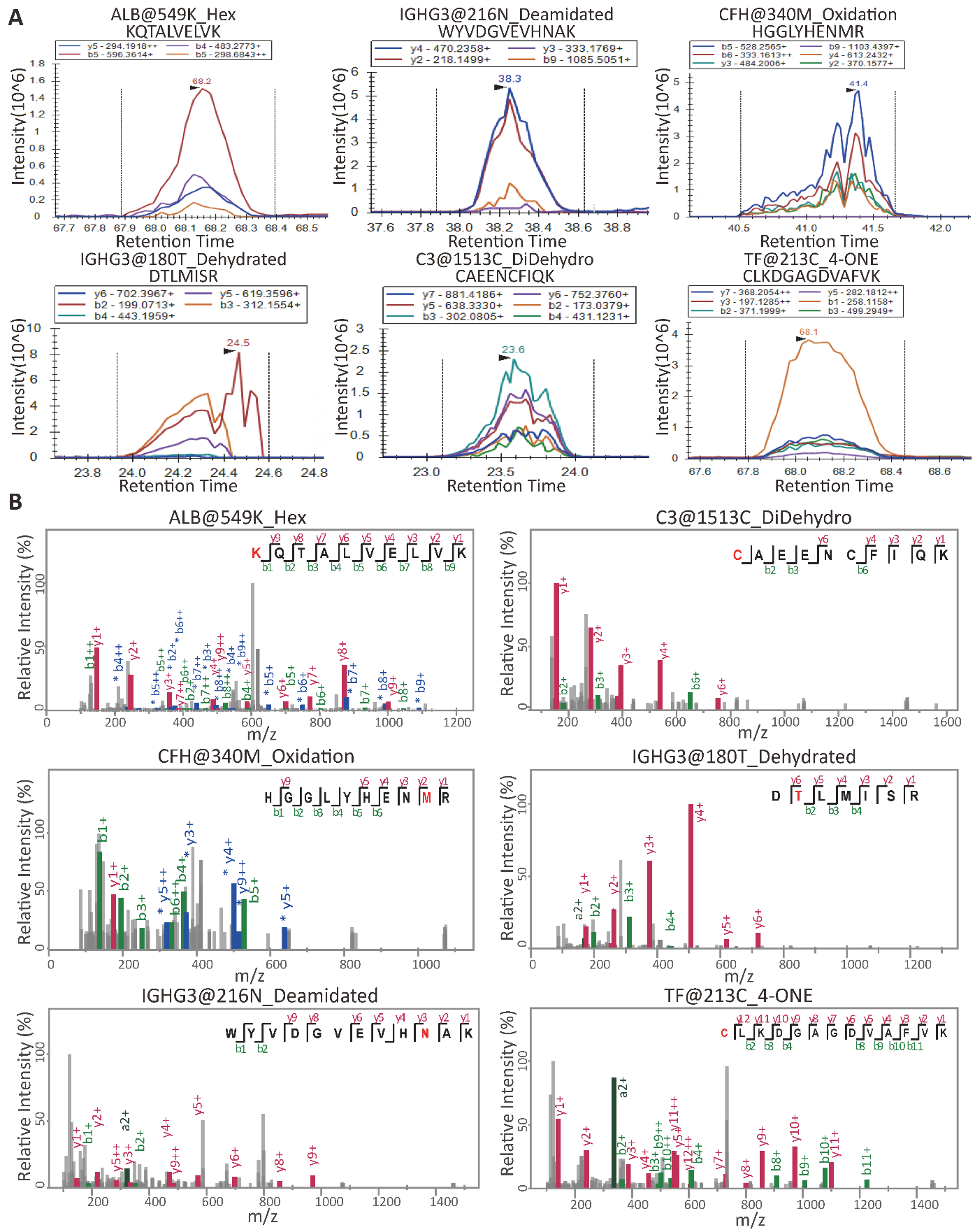
**Fig. S7. Differential abundance of ncAA-modified signature peptides across COVID-19 disease states.**

1. Corresponding representative ion chromatograms for Parallel Reaction Monitoring (PRM) quantificating signature ncAA-modified peptides across COVID-19 disease state.
2. Annotated MS/MS spectra for representive ncAA-modified peptides in Figure 4C,4D.


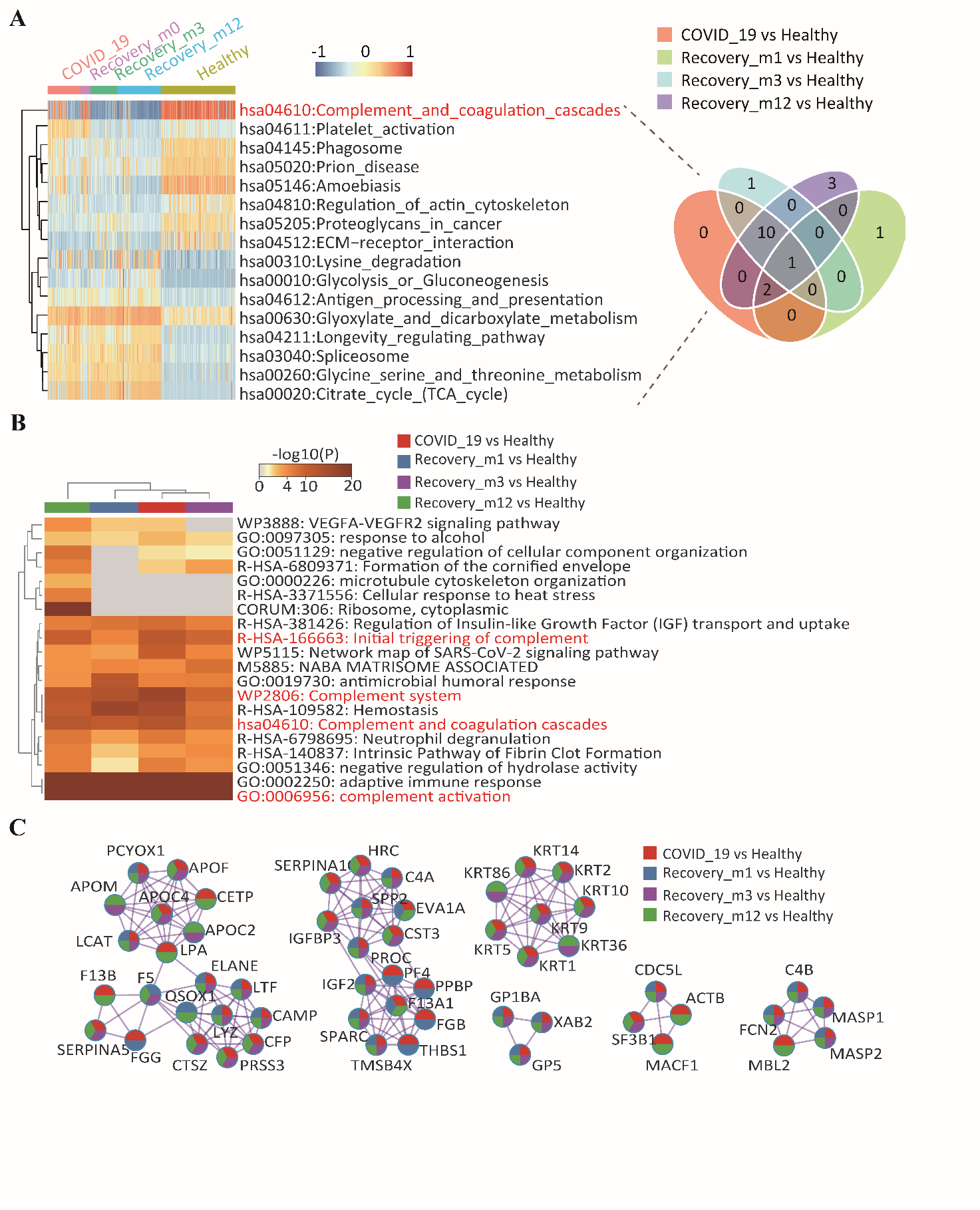


**Fig. S8. Quantitative analysis of serum Proteomic profile.**

**(A**) Heatmap showing the different enriched KEGG pathways identified by KEGG dataset-based Gene Set Variation Analysis (GSVA) of proteomic profile.

**(B**) Enrichment analysis of different expression proteins (DEPs) identified by contrasting the disease and recovery stages of COVID-19 to healthy populations. (Cutoff: |fold change| > 2, FDR < 0.05)

**(C**) The protein-protein interaction network (PPI) of DEPs identified in each contrasts.


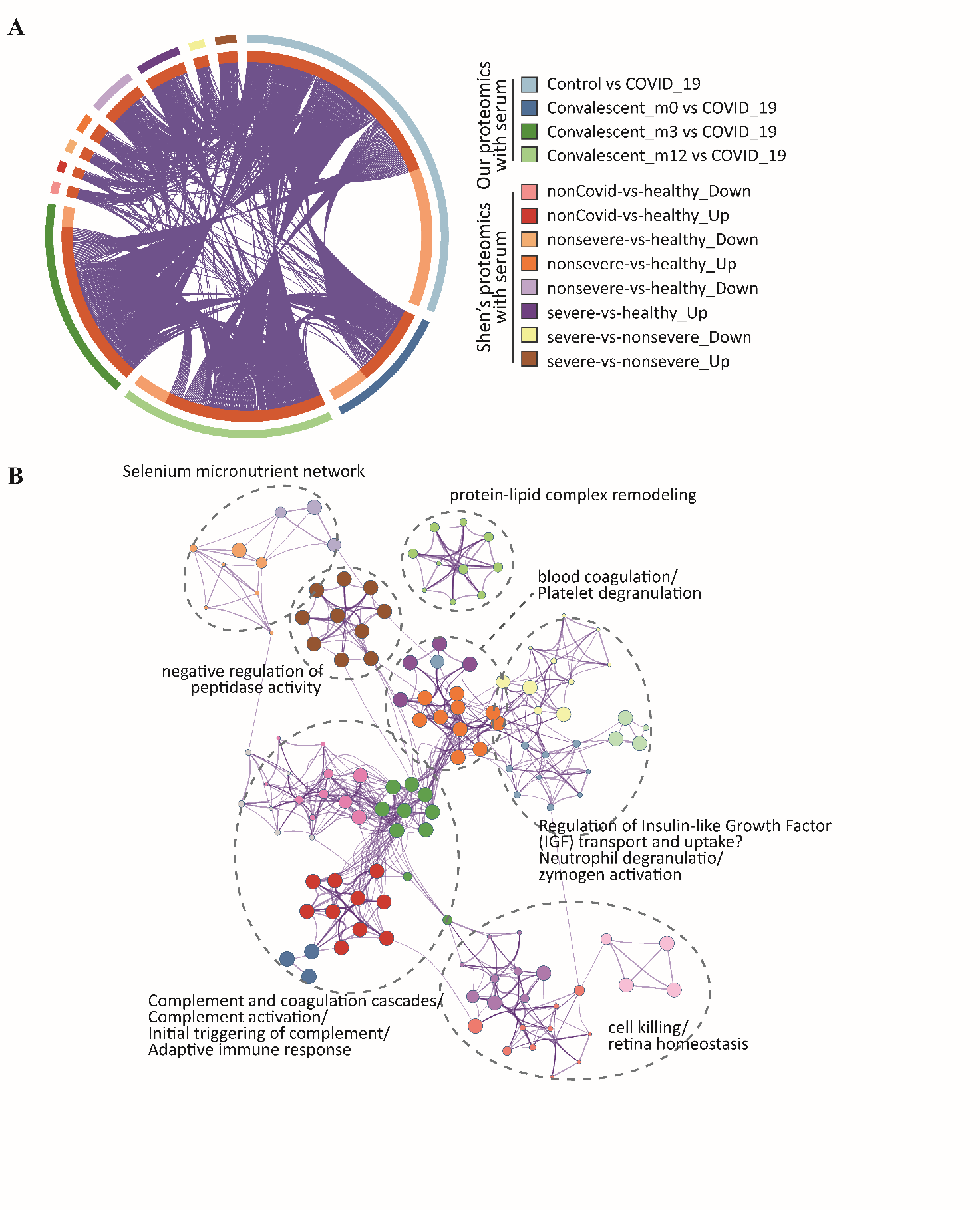


**Fig. S9. Comparative analysis of serum proteomics from different COVID-19 datasets.**

**(A**) Circos diagram showing the overlap of differentially expressed proteins identified by different datasets.

**(B**) Enrichment analysis of all different expression proteins identified in different datasets.


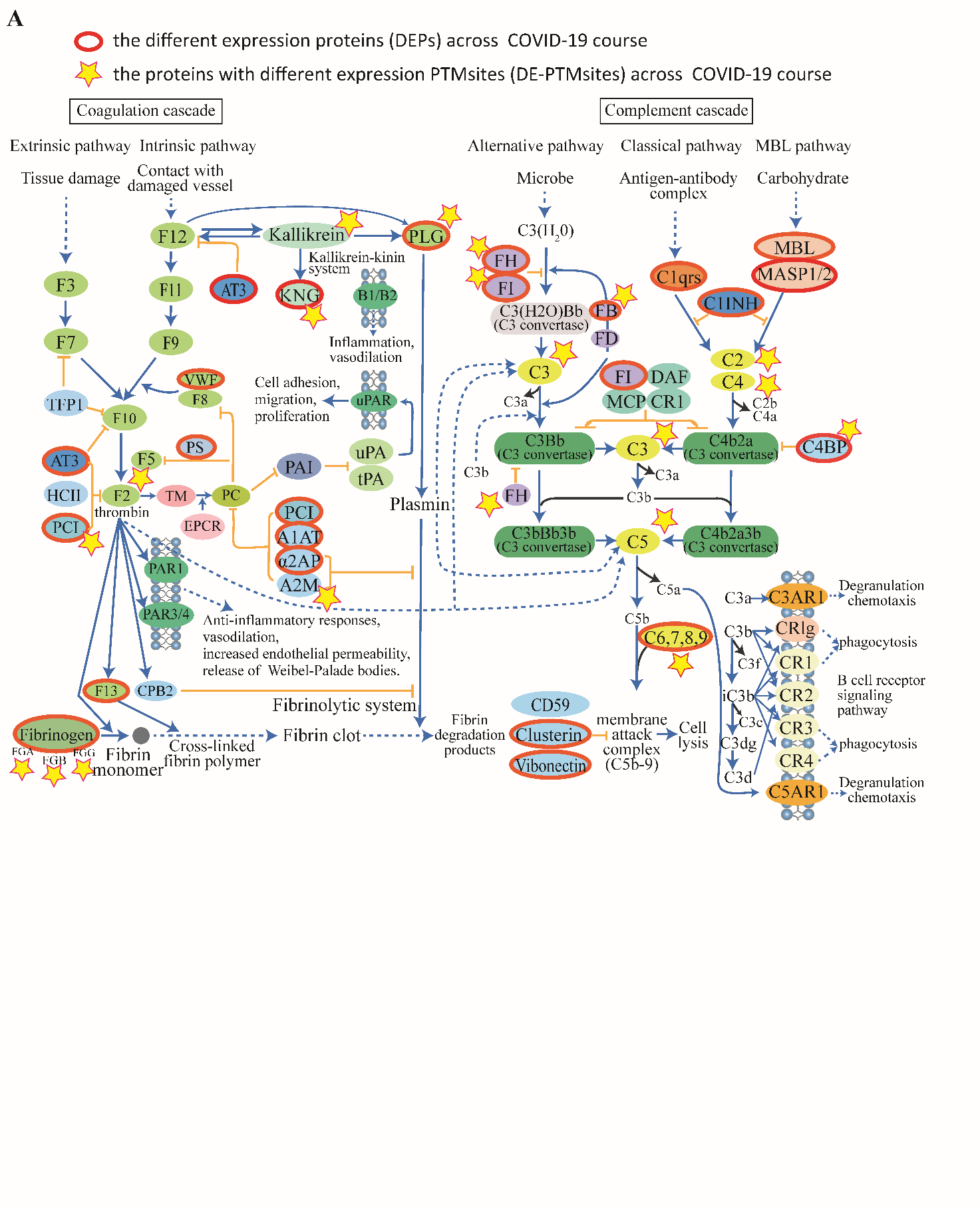


**Fig. S10 Schematic diagram of differentially expressed proteins (red circle) or proteins with differentially expressed ncAAs (yellow star) in the complement and coagulation cascades.**


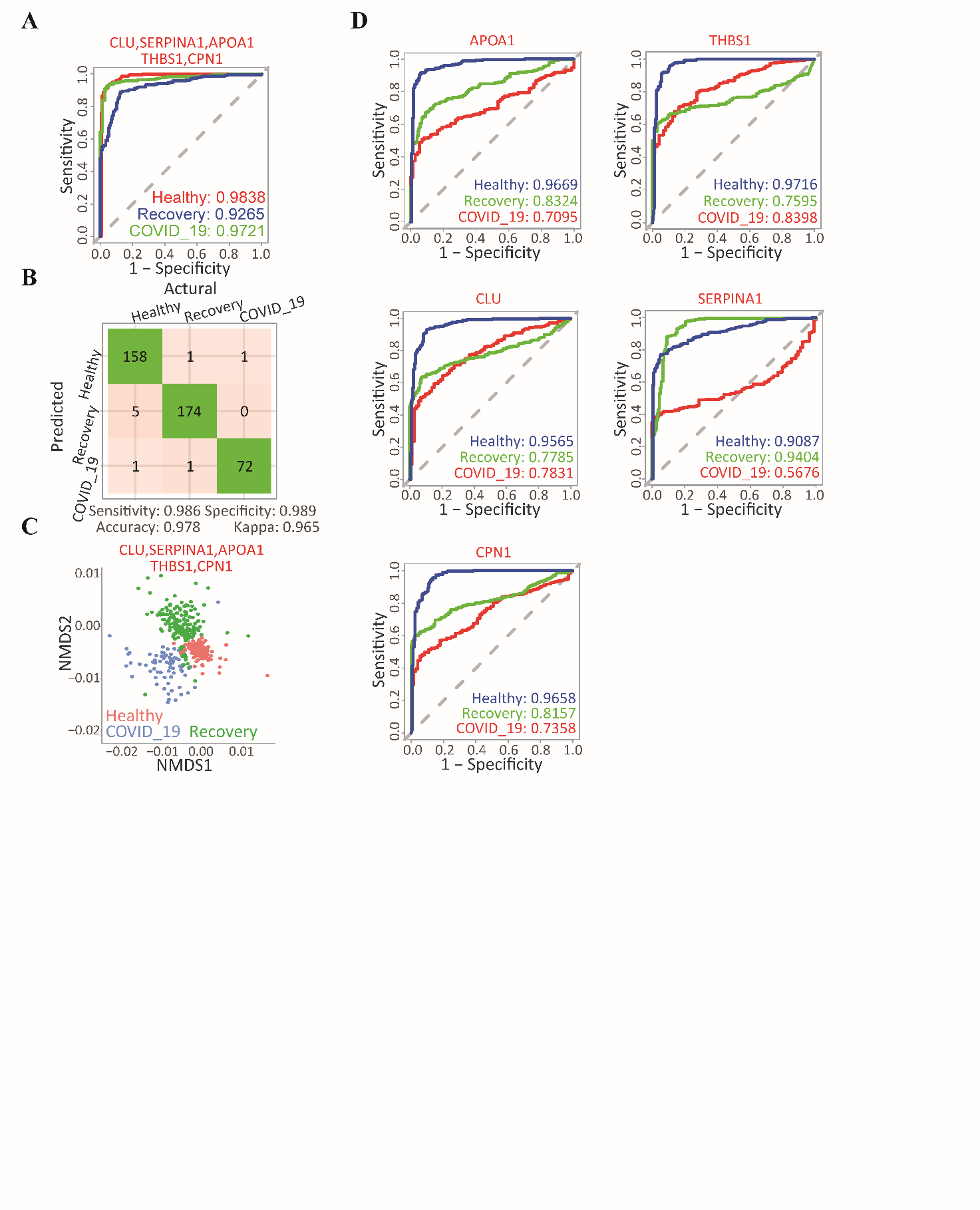


**Fig. S11. Machine-learning strategy identifies the representative proteins in acute and recovery stages of COVID-19 patients.**

**(A, B, C**) ROC curve **(A)**, confusion matrix **(B)**, NMDS assay **(C)** of indicated proteins combinations for classing the disease and recovery stages in COVID-19, and healthy populations in the cohort 1 and 2 combinations.

**(D)** ROC curve of indicated proteins for classing the disease and recovery stages in COVID-19, and healthy populations in the cohort 1 and 2 combinations.
